# Exploring levels of wellbeing and Physical Activity (PA) in UK hypermobile females 18-70

**DOI:** 10.1192/j.eurpsy.2026.10768

**Published:** 2026-07-31

**Authors:** L. A. Mcpherson, A. Hughes, I. Campbell

**Affiliations:** Psychological Sciences for Health, University of Strathclyde, Glasgow, United Kingdom

## Abstract

**Introduction:**

Hypermobility (HM) is a heritable connective tissue difference found in 20% of adults, disproportionately females (70%). The 2017 nosology distinguishes Generalised joint HM (GJH), HM Spectrum disorder (HSD) and HM Ehlers Danlos (hEDS). Emerging evidence suggests up to half of HM are also neurodivergent (ND), yet the intersection between HM and ND is rarely explored. This comorbidity likely confounds population-based studies over all age groups on anxiety, depression, stress, quality of life and levels of PA and Sedentary Behaviour (SB).

**Objectives:**

We examined (1) levels of anxiety, stress, depression and quality of life in people with HM. (2) objectively measured PA in relation to WHO guidelines and SB; (3) differences across HM classification, presence of ND and diagnostic status.

**Methods:**

Mixed methods: 2 phases: Phase 1 online questionnaire (n =110; age 18-59 (M =35.8 years) including DASS8, QLES-SF with extended qualitative response, Phase 2 accelerometers n= 19 for PA and SB.

**Results:**

**Mental Health:** 70% diagnosed/self-diagnosed ND; 62.7% with long-term conditions. ND hypermobile (NDH) report greater moderate to severe levels of Depression (U=939*, p*=.029), Anxiety (U=910, *p* = 0.018), Stress (U = 683.5, *p* = < .001). ND status associated with higher risk of pain severity, psychiatric comorbidity, and multiple medications (*U*=795.5 *p*=<.0019).

**Quality of Life:** hEDS participants reported significantly lower QoL compared to GJH (adj. p=.0119) and HSD (adj. p=.030). Thematic analysis identified five key themes: (1) inextricable body–brain connection, (2) unpredictability and disruption, (3) invisibility and invalidation, (4) uncertain future, and (5) resilience despite adversity.

**PA:** 84.2% (16/19) met 150 min/week MPA guideline; only 36.8% (7/19) met the extended 300 min/week recommendation relative to sedentary time. Self-reported PA significantly underestimated objectively measured PA (t(11)=4.47, p<.001, r=.819). Qualitative comments revealed perceived limits of participation (“I want to do more but cannot increase this without long periods of pain and recovery”).

**Conclusions:**

We provide novel evidence of the high comorbidity of HM and ND and its significant impact on mental health, pain, and medication burden. While objective PA levels suggest most participants meet minimum WHO guidelines, elevated SB and underreporting of PA highlight unique barriers and negative self-concept in hypermobile women. QoL lowest in hEDS, explained partly by the unpredictable and invisible nature of symptoms. These findings suggest PA alone may not protect mental health in this group, and that diagnostic and neurodivergent status are important moderators of wellbeing outcomes. Recognition of HM–ND comorbidity is critical for developing targeted interventions and improving clinical support.

**Disclosure of Interest:**

None Declared

